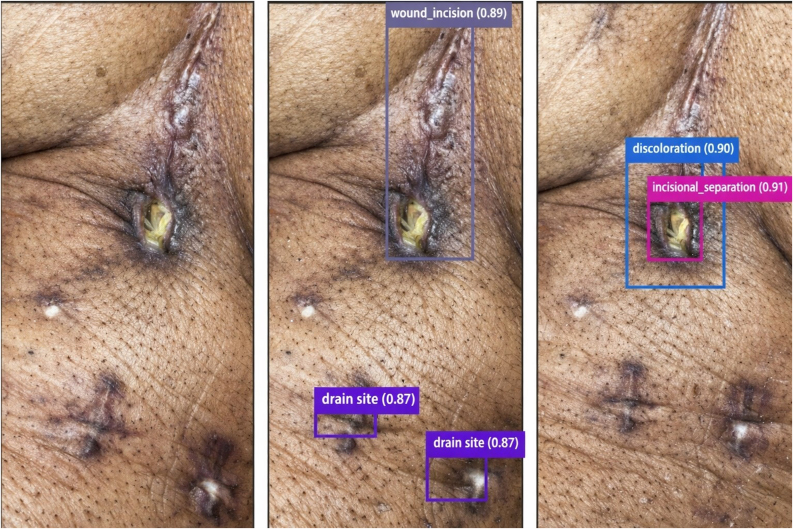# AI-enabled digital wound monitoring after cardiac surgery: a randomised controlled feasibility, safety, and acceptability trial^☆^

**DOI:** 10.1016/j.jhin.2026.04.020

**Published:** 2026-08

**Authors:** M. Rochon, J. Tanner, K. Cariaga, J. Jurkiewicz, J. Beckhelling, R. Harris, K. Wilson, L. Dhoonmoon, S. Bolton, J. Bouttell, D. Davis, A. Shipolini, R. Magboo, F. Oezalp, V. Chester

**Affiliations:** a Guy's and St Thomas' NHS Foundation Trust, London, United Kingdom; b University of Nottingham, Nottingham, United Kingdom; c Isla Care Ltd, London, United Kingdom; d Derby Clinical Trials Support Unit, Derby, United Kingdom; e NIHR Research Support Service (RSS) Hub Delivered by the University of Leicester and Partners, Nottingham, United Kingdom; f Liverpool Heart and Chest Hospital NHS Foundation Trust, Liverpool, United Kingdom; g London North West University Healthcare, London, United Kingdom; h Centre for Healthcare Equipment and Technology Adoption (CHEATA), Nottingham, United Kingdom; i Barts Health NHS Trust, London, United Kingdom; l The Newcastle Upon Tyne Hospitals NHS Foundation Trust, Newcastle, United Kingdom

**Keywords:** Randomised controlled trial, Artificial intelligence, Skin tone, Surveillance, Wound infection

## Abstract

**Background:**

Surgical site infection after cardiac surgery is a common cause of morbidity and unplanned healthcare use, with most infections developing after hospital discharge. Remote wound monitoring using smartphone technology and artificial intelligence (AI) may support earlier identification of complications.

**Aim:**

To evaluate the feasibility, acceptability, and safety of an AI-enabled digital wound monitoring platform plus usual care (Isla-AI) compared with usual care (UC) alone.

**Design, setting, and participants:**

This multi-centre, two-arm randomised controlled feasibility trial was conducted at two U.K. hospitals between August 2024 and January 2025. Adults undergoing cardiac surgery were randomised to receive Isla-AI or UC. The study was not powered to assess effectiveness.

**Results:**

120 patients were randomised and participated (Isla-AI *N* = 62; UC *N* = 58). Feasibility targets were exceeded: 60% of eligible patients approached consented, 95% of Isla-AI participants submitted at least one image, and 92% completed the study. Ninety-eight percent of images were suitable for clinical assessment. Clinician agreement with AI priority flags was 87%. AI prioritisation performance was slightly better for patients with darker skin tones. More than half of participants required assistance to capture or submit wound images. Patient- and staff-acceptability of AI was largely favourable. Adverse and serious adverse event rates were similar across both groups. The proportion of patients accessing National Health Service (NHS) resources for wound-related problems and antibiotics was lower in the Isla-AI group.

**Conclusions:**

These findings support progression to a large, definitive multi-centre effectiveness trial, with further attention to equity, usability, and workflow integration.

**Trial registration:**

IRAS 338141; local project UHDB/2022/024. ISRCTN16900119.

**Clinicaltrials.gov:**

NCT06475703. Date registered: 20/06/2024.

## Introduction

Surgical site infections (SSIs) remain among the most common postoperative complications and are associated with increased morbidity, unplanned healthcare use, and substantial costs to health systems [[1], [2], [3], [4]]. Early identification and timely management are critical; however, postdischarge surveillance is not routinely undertaken, and when performed, it is traditionally retrospective [5] and does not directly benefit patient care [6]. As lengths of hospital stay continue to shorten, there is growing interest in remote postoperative monitoring in real time to support early detection of wound complications.

**Table I:** Summary of baseline data

	Isla-AI *N* = 62	UC only *N* = 58
**Age (years) -***mean (95% CI)*	*64.7 (62.7 to 66.7)*	*63.6 (61.3 to 66.0)*
**BMI -***median (IQR)*	26.6 (24.3–29.4)	28.7 (25.7–31.4)
**Sex** n (%)		
**Male**	54 (87.1)	50 (86.2)
**Female**	8 (12.9)	8 (13.8)
**Intersex**	0 (0)	0 (0)
**Diabetes mellitus**	28 (45.2)	23 (39.7)
**Current smoker**	7 (11.3)	4 (6.9)
**Relevant disability**^a^*N* (%)		
**Visual impairment**	7 (11.3)	6 (10.3)
**None**	0 (0)	0 (0)
**Ethnicity***N* (%)		
**White**	36 (58.1)	39 (67.2)
**Asian**	18 (29.0)	15 (25.9)
**Black**	2 (3.2)	0 (0.0)
**Other/mixed**	4 (6.5)	3 (5.2)
**Not stated/unknown**	2 (3.2)	1 (1.7)
**Category of index of multiple deprivation(11) (deciles: 1 = most deprived)***N* (%)		
**More deprived (deciles 1–5)**	30 (48.4)	26 (44.8)
**Less deprived (deciles 6–10)**	29 (46.8)	30 (51.7)
**Not known**	3 (4.8)	2 (3.5)
**Lives alone**	4 (6.5)	14 (24.1)
**Light skin tone** (Ho and Robinson skin tone scale 1) (12) *N* (%)	40 (64.5)	40 (69.0)
**Dark skin tone** (Ho and Robinson skin tone scales 2–6) (12) *N* (%)	22 (35.5)	18 (31.0)
**English speaking** n (%)		
**Yes**	57 (91.9)	51 (87.9)
**No**	5 (8.1)	7 (12.1)

a All cases were visual impairment. None recorded for manual dexterity or cognitive impairment. BMI, body mass index; CI, confidence interval; IQR, interquartile range; UC, usual care.

Digital surgical wound monitoring is well-liked by patients and engagement is high [7,8]. Evaluations suggest wounds are identified earlier and unplanned healthcare use is reduced. [9] Identifying wounds early enables them to be managed before they worsen and become harder and more expensive to treat [10]. However, implementing digital monitoring at scale creates an additional staffing challenge [11,12]. This additional staffing challenge can be addressed through artificial intelligence (AI). AI offers an opportunity to support digital wound monitoring and facilitate its implementation at scale and pace. AI advances, particularly in image-based analysis, provides a novel approach to postdischarge surveillance by enabling automated identification of features associated with infection and impaired healing. By supporting prioritisation of cases requiring clinical review, AI-enabled monitoring can assist staff to manage their workload. [12]. This scalable approach represents a promising intervention that warrants prospective evaluation. Despite this potential, there is limited evidence of the implementation of AI-supported monitoring pathways, particularly in relation to patient engagement, data quality, and integration into clinical workflows. A feasibility study therefore is needed to assess acceptability, safety and practicality of data collection prior to a definitive effectiveness trial.

The aim of the study was to evaluate the feasibility, safety and acceptability of using AI to prioritise images with a wound healing concern for clinician review and to test tools for the collection of resource use.

## Methods

### Study design

This unblinded, parallel-group randomised feasibility trial was conducted at two cardiac centres. St Bartholomew's Hospital, London and the Freeman Hospital, Newcastle, UK were selected as they represent urban and rural/coastal locations with a diverse patient ethnic demographic. Patients having coronary bypass graft (CABG) surgery without a pre-existing wound infection were eligible to participate. Smartphones and internet access were offered if required. Recruiting 60 patients to both groups in this feasibility study allowed us to estimate recruitment and drop-out rates with a 95% confidence interval (CI) of within ± 10% even if the rates were 50% for these two feasibility outcomes. Initial screening was presurgery, with final eligibility assessed after surgery. Screening and consenting were carried out by the usual care (UC) team. Randomisation was implemented using the electronic data capture system used by the study clinical trials unit. Participating patients were randomised at enrolment using 1:1 stratified randomisation with mixed block sizes stratified by centre and sex, to receive either usual postoperative wound follow-up care only (UC) or usual follow-up wound care plus digital wound monitoring (Isla) with AI (Isla-AI). Data were collected at baseline, and 30 days and 60 days after surgery through surveys, interviews, case-note review, platform review and phone calls.

#### Isla-AI

Participants allocated to Isla-AI received digital postoperative wound monitoring via a secure platform. Wound images and completed wound healing questionnaires were submitted in response to scheduled requests on days 7, 14, and 21 postsurgery. The AI model analysed submitted images to identify visual features associated with infection or impaired healing and assigned a priority flag to support clinician triage (Supplementary Material). All submissions were reviewed by trained clinicians, with AI used to prioritise cases requiring more urgent attention rather than diagnosing an infection.

#### UC

Participants allocated to UC received standard postoperative follow-up according to local practice, which could include outpatient or virtual consultations, advice to contact primary or secondary care services, or routine surgical site infection surveillance at 30 days.

#### Primary outcomes

• Feasibility including:
  o Recruitment rate (proportion of eligible patients consenting)
  o Retention (study completion)
  o Adherence to the intervention (full adherence: all three time points; partial adherence: one or two time points)
  o Prespecified progression criteria: ≥25% of eligible patients consent; ≥80% of Isla-AI participants submit ≥1 image; ≥60% of participants complete the study
• Safety was included in the clinical usability of wound images and adverse events (AEs) and serious adverse events (SAEs).
• Acceptability was assessed via patient and staff surveys, including satisfaction, burden, confidence, and usability (including need for assistance) and staff workload.

#### Secondary outcomes

• Clinician agreement with AI prioritisation
• AI performance (correct classifications, false positives, and false negatives)
• Time to clinician response (from submission to review; summarised at 24 and 48 h)
• Clinical outcomes including SSI at 30 days and wound healing without complications at 30 and 60 days
• Healthcare resource use (planned and unplanned care, emergency attendance, re-admissions, antibiotic use, and patient-incurred burden)

#### Equity and subgroup analyses

Prespecified analyses assessed AI performance by skin tone [13], with skin tone 1 grouped as ‘light’ and skin tones 2–6 grouped as ‘dark’, based on expert opinion and to align with prior skin tone descriptors for the AI. Agreement and error rates were compared across groups. Digital inclusion measures included need for assistance and usability barriers.

#### Data collection

Data were collected at baseline, 30 and 60 days via surveys, telephone follow-up, platform data, and case note review. Platform-derived metrics (including response times) were extracted from system records.

#### Statistical analysis

Analyses followed the prespecified Clinical Investigation Plan and were primarily descriptive. AI agreement was summarised as concordance; error types were reported; and subgroup analyses by skin tone were descriptive**.** Analyses were conducted using Stata v18.

### Patient and public involvement

Patient and public involvement (PPI) and equality, diversity, and inclusion (EDI) leads were integral to the study management team. PPI group members were involved throughout the study and ensured the study remained patient-centred, supported software design and evaluation, reviewed protocols and patient-facing documents, and provided input on reporting and dissemination.

## Results

### Feasibility

#### Recruitment and attrition

**Figure 1:**
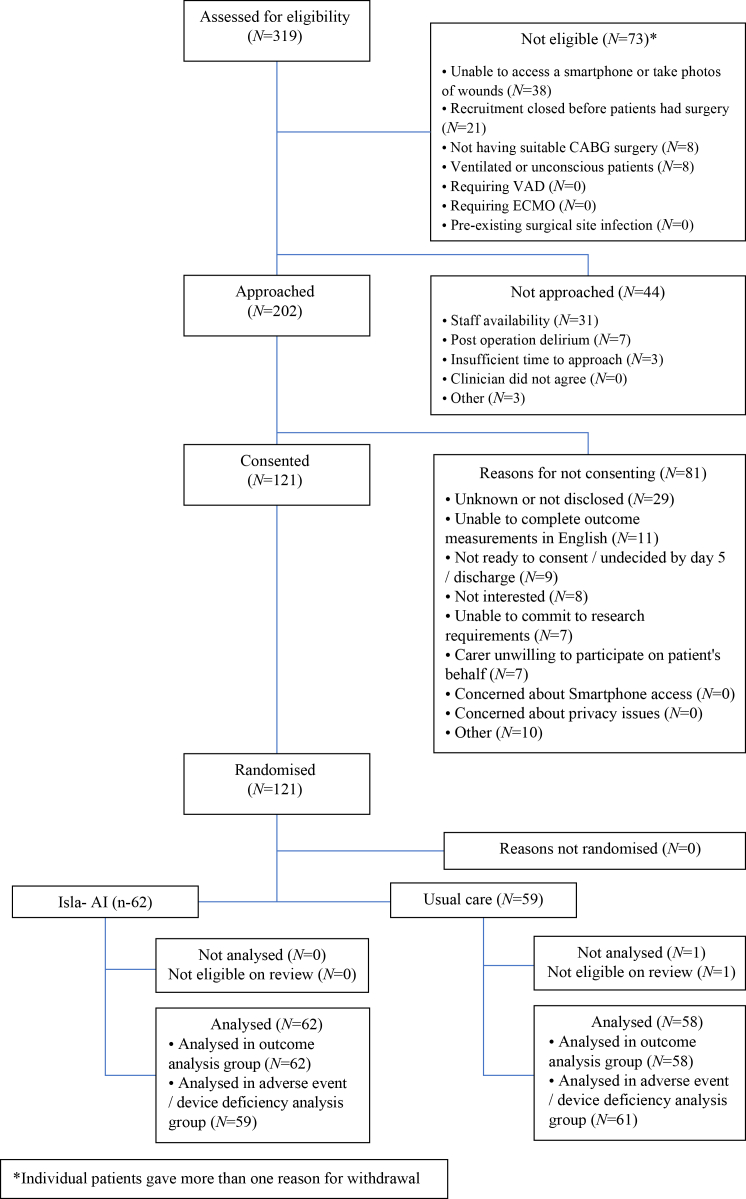
CONSORT diagram

Recruitment ran from 01 August 2024 until 31 January 2025. Three hundred and nineteen patients undergoing CABG surgery during the 6-month recruitment period were screened for eligibility (Figure 1). Two hundred and forty-six patients (77%) were considered eligible, and 73 patients were considered ineligible. Reasons for ineligibility are listed on Figure 1. Of the 246 eligible patients, 202 patients were approached until the required sample size of 121 patients consented to participate, a recruitment rate of 60%. Sixty-two participants were randomised to Isla-AI and 59 to UC, though one patient in the UC group was considered ineligible on review due to having undergone inappropriate surgery and was withdrawn, leaving 58 patients in the UC group. One hundred and ten of the 120 randomised, eligible participants (92%) completed the study; 58/62 (94%) in Isla-AI and 52/58 (90%) in UC. Ten patients (4 Isla-AI and 6 UC) were withdrawn before the end of the study. The most common reason was loss to follow-up (3 Isla-AI patients, 5 UC patients). Three patients in the Isla-AI group who did not submit any images were analysed for clinical outcomes in the Isla-AI group and were analysed in the UC group for AEs and device deficiency as per the Clinical Investigation Plan.

#### Participant characteristics

Overall, both treatment groups were similar in terms of key demographics, operative details, socioeconomic status, ethnicity, and skin tone (Table I), although patients in the UC group were more likely to live alone than those in the Isla-AI group (7% Isla-AI patients, 24% UC).

#### Progression criteria

All progression criteria were met; 59% of eligible patients who were approached consented (target 25%), 95.2% of patients in the Isla-AI group adhered to the intervention (target 80%), and 93% of the Isla-AI participants completed the study (target 60%).

#### Patient adherence to digital platform requests to submit data

Patients in the Isla-AI group received requests to submit data each week for three weeks. For the Isla-AI group, 95% of patients (59/62) submitted on at least one occasion. Seventy-seven percent of participants (48/62) demonstrated full adherence by submitting during each of the three weeks and 18% (11/62) were partially adherent. Of the three patients who did not submit any images, one patient did not receive any image submission links, and no reasons were documented for the remaining two non-submitting patients. The image submission response rate in week 1 was 95%% (59/62), 94% (58/62) in week 2, and 77% (48/62) in week 3.

##### Acceptability

###### Patient satisfaction with Isla-AI (Isla-AI group only)

Fifty-three out of 62 patients in the Isla-AI group completed an online satisfaction survey at 30 days. Patient-reported satisfaction with Isla-AI was high: 83% agreed or strongly agreed that they liked using the Isla platform (74% and 9%) and 90% of patients were confident that Isla-AI worked (81% agreed, 9% strongly agreed). The perceived burden of taking photos and responding to information requests was low: 57% and 34% of patients agreed that responding to requests for information required ‘little effort’ or ‘no effort’, with 94% of patients saying it did not interfere with undertaking other daily priorities. The platform's monitoring schedule was well received: 91% rated weekly prompts for 30 days as ‘just right’, with 8% preferring more frequent prompts; 75% judged the 30-day duration of follow-up as ‘just right’, while 21% considered it too short. Many participants reported they required assistance: 59% to take photographs and 66% to submit photographs/questionnaires. Despite this, adherence was high; only 17% reported at least one missed submission, most commonly due to forgetting or helper unavailability. Approximately one-third of participants reported at least one usability issue, including ability to use technology (*N* = 9), difficulty taking photos unaided (*N* = 5), or understanding English (*N* = 4).

###### Staff satisfaction with Isla-AI

Seven nursing or surgical staff who participated in the study completed the survey assessing acceptability, perceptions of functionality, and ease-of-use. Staff roles are withheld to preserve anonymity. Staff undertook varied digital monitoring tasks, including reviewing images. Median time spent on digital monitoring or study activities related to digital monitoring was 2 h per week. A range of 1–22 h was reported with six staff saying this was up to 8 h per week in addition to existing duties and one staff member citing 22 h a week.

Staff attitudes towards the platform were favourable: ‘liked using the platform’ split between agree (*N* = 3) and strongly agree (*N* = 3) with one neutral; ‘acceptable to use’ strongly agree (*N* = 3), agree (*N* = 2), neutral (*N* = 2). Confidence that the platform worked: disagree (*N* = 1), agree (*N* = 3), strongly agree (*N* = 2). Five staff did not currently monitor wounds remotely, yet six felt their hospital was mostly ready to implement remote monitoring. Comfort was high for using AI to triage images of wounds that appear infected (very comfortable *N* = 4, slightly *N* = 2, not comfortable *N* = 1), but slightly lower for using AI to diagnose wound infections (very comfortable *N* = 1, slightly *N* = 4, not comfortable *N* = 1, no opinion *N* = 1).

All surveyed staff endorsed monitoring all postoperative patients, typically starting 1–7 days postoperative and stopping at 10–60 days (*N* = 6) or when healed (*N* = 1). Proposed frequency of monitoring ranged from every 2–3 days to weekly (*N* = 4), with escalation if concerns arose.

###### Patient satisfaction with follow-up care

Patients in both groups rated their follow-up care on a scale of one to five, with one the lowest rating and five the highest. Approximately three-quarters of patients gave a rating of three, four, or five for their follow-up care (29% rated it at three, 27% at four, and 25% at five). Within the treatment groups, there was a slight tendency for Isla-AI patients to rate follow-up higher than UC-only patients. Around one-quarter of Isla-AI patients (26%) gave their follow-up care the middle score of three vs 32% of UC patients, whereas 34% of Isla-AI patients scored follow-up at four (a better score than three) than just 19% of UC patients. For the highest rating of five, the scores were similar, at 26% of Isla-AI patients than 23% of UC patients. Sixteen patients rated their care at the lowest level, six in the Isla-AI group (11%) and 10 in UC (21%).

### Safety

#### Wound image quality

A quality image was defined as an image with sufficient focus, lighting, and field of view to permit clinical assessment as determined by the reviewing clinician. Wound image quality was high, 97.7% (349/357) images submitted were suitable for clinical decision-making regarding wound healing. Three of the eight unsuitable images were blurred and five images, taken by three participants, did not meet the criteria (two patients sent photos of wounds with dressings on and one patient sent photos of a printout given to them with photos of their wound taken at hospital at the time of discharge).

#### Clinician agreement with Isla-AI algorithm priority flags

A total of 349 wound images were reviewed by the Isla-AI model and the clinicians. Agreement between the AI model and clinical assessment was 87.4% (305/349). The Isla-AI algorithm failed to identify five images that required priority review and incorrectly prioritised 39 images that did not require priority review. Two hundred and thirty-nine images were from patients with a light skin tone and 110 images were from patients with a darker skin tone. Agreement for images of a light skin tone was 84% (200/239), while agreement for images of a darker skin tone was 95% (105/110).

#### Time to respond to patient submitted data

Clinicians responded to 70% of patient-submitted data within 24 h and 86% of submissions were responded to within 48 h. Of the 154 images flagged for priority review by Isla-AI, 116 (75.3%) were responded to within 24 h.

#### Adverse events

Thirty-three patients in the Isla-AI group reported 46 AEs and 36 patients in the UC group reported 48 AEs. Examples of AEs included infections, chest pain, leg swelling, and vomiting. Three patients were recorded as having four SAEs. In the Isla-AI group, one patient experienced chest pain, and another patient experienced deep vein thrombosis postoperatively and pulmonary embolism. One patient in the UC group had an SSI recorded by clinicians as a serious adverse event. No suspected unexpected serious adverse reactions were reported.

#### Clinical outcomes

The SSI rate at 30 days for the Isla-AI group was 11.5% (7/61) and 16% (9/55) in the UC group. The wound healing rate without complications at 30 days was 81% (48/59) in the Isla-AI group and 73% (38/52) in the UC group, with most wounds healing by 60 days (90% Isla-AI, 79% UC).

#### Resource use

The proportion of patients having planned follow-up care appointments (GP appointments, outpatient clinic visits, and district nurse visits) in the first 30 days postsurgery were similar for both groups (61% Isla-AI vs 57% UC). Unplanned care visits relating to wounds were accessed by a smaller proportion of patients in the Isla-AI group (47%) compared with 60% of patients in the UC group.

Two patients in the Isla-AI group and three patients in UC attended the Emergency Department, one patient in the Isla-AI group was re-admitted due to their wound, no patients in either group had further surgery, and 19% of patients in the Isla-AI group were prescribed antibiotics for their wound compared with 28% in the UC group. Estimated travel distances for both groups were similar and no patients reported having to take extra time off work because of their wound, as most patients were retired or still on sick leave due to the operation.

Resource use was more costly in the Isla-AI arm, mainly due to the one patient who was re-admitted for a long stay and incurred significant treatment costs.

#### Data not reported

Patient and staff acceptability data obtained through qualitative interviews, and health economic modelling will be reported elsewhere. Days to diagnosis of infection could not be determined as, at the 30-day assessment, patients could not reliably recall exactly when an infection had been diagnosed.

## Discussion

This multi-centre randomised feasibility trial demonstrates that postdischarge digital wound monitoring with AI after cardiac surgery is feasible, safe, and acceptable, with high rates of clinically usable imaging and strong participant engagement. These results justify a prospective, large-scale trial for external validation of Isla-AI. This study adds prospective evidence that AI-enabled wound monitoring can be implemented in routine care, with reliable patient data capture and integration into clinical workflows, including across different skin tones.

Engagement with the Isla-AI platform was exceptionally high, with 95% of participants submitting at least one wound image and 77% demonstrating full adherence to all scheduled requests for information. This level of adherence exceeds that reported in most prior studies of AI-supported or smartphone-enabled SSI surveillance [9], possibly reflecting patient-friendly design features. [7] Other digital SSI surveillance studies report a much shorter postsurgery follow-up period [14], or do not capture engagement. [15].

Agreement between AI-generated priority flags and clinician assessment was high (87.4%) and compares favourably with performance reported in other image-based AI SSI studies. [15,16] Notably, only one confirmed SSI was not flagged for priority review. Ongoing model refinement aims to enhance AI performance for surgical wound complication detection while improving sensitivity in darker skin tones (NIHR501558). Similar to a large US study [15], this study focused AI training on wound images alone to maximise label quality and generalisability across incision types. Within this context, previous work by McLean *et al.* [9] has demonstrated that adding wound images to symptom-based models can substantially improve specificity, supporting a multi-modal approach. Taken together, our findings suggest that while image-only AI can support safe prioritisation, integration with patient-reported symptoms represents the next step for improving performance and reducing unnecessary escalation.

Several lessons were identified that are important for future implementation and trial design. First, although adherence was high, more than half of the participants required assistance to capture or submit images, highlighting the need to design inclusive pathways and provide support to minimise digital exclusion. Second, while AI prioritisation showed high agreement with clinicians, the presence of false positives and occasional missed cases underscores the importance of maintaining clinician oversight and refining model performance. Third, integration into clinical workflows requires consideration of staff time and resource implications.

This is among the first prospective studies globally to evaluate an AI-assisted remote SSI surveillance pathway. A key strength of this feasibility study is the prospective assessment of equity and usability in AI-enabled postoperative surveillance. Over one-third of participants in the Isla-AI group had darker skin tones, and algorithm prioritisation performance was slightly better in patients with darker skin tones. This is notable given that prior studies in chronic wound monitoring have suggested image-based algorithms may perform differently across skin tones, potentially exacerbating disparities if not carefully evaluated. [17] The Fitzpatrick skin type scale, commonly used in retrospective analyses and secondary datasets, was originally developed to classify skin response to ultraviolet exposure rather than visible skin appearance. [18] In this study, we therefore prospectively used the Ho and Robinson skin tones tool [19], which captures gradations in skin appearance, enabling more consistent point-of-care classification and more robust equity-focused assessment of AI performance.

Although SSIs, and the proportion of unplanned resource use and antibiotic use, were lower in the Isla-AI group, the Isla-AI group was more costly, mainly due to one patient re-admission; however, changes to the delivery pathway would make digital monitoring with AI cost-effective.

As with much of the existing AI SSI surveillance literature, limitations include the smaller sample size, and centre-specific workflows. However, this highlights the importance of moving beyond retrospective evaluations. Although fewer SSIs were observed in the Isla-AI group, this feasibility study was not designed or powered to assess the effectiveness of the intervention. Few prior AI-enabled SSI surveillance studies have prospectively evaluated equity, skin tone representation, or need for assistance. Although no major safety signal was identified, under-representation of darker skin tones and frequent need for assistance underscore the importance of deliberate equity-focused design in future trials.

In conclusion, this multi-centre randomised feasibility trial, postdischarge digital wound monitoring with Isla-AI was feasible, acceptable, and safe after cardiac surgery. Recruitment, adherence, and study completion exceeded *a priori* thresholds. Most participants supplied clinically usable images across all requested time points, and patient survey responses indicated high acceptability and confidence in the approach. Clinician disagreement with Isla-AI prioritisation predominantly reflected conservative ‘healing concern’ flags, and no suspected unexpected serious adverse reactions occurred, supporting the safety of remote monitoring within a supervised clinical pathway.

## CRediT authorship contribution statement

**M. Rochon:** Writing – review & editing, Writing – original draft, Methodology, Funding acquisition, Formal analysis, Conceptualisation. **J. Tanner:** Writing – review & editing, Writing – original draft, Methodology, Funding acquisition, Conceptualisation. **K. Cariaga:** Writing – review & editing, Visualisation, Resources, Project administration. **J. Jurkiewicz:** Writing – review & editing, Software, Methodology, Investigation, Funding acquisition. **J. Beckhelling:** Writing – review & editing, Methodology, Funding acquisition, Formal analysis. **R. Harris:** Writing – review & editing, Methodology, Funding acquisition. **K. Wilson:** Writing – review & editing, Funding acquisition. **L. Dhoonmoon:** Writing – review & editing, Funding acquisition. **S. Bolton:** Writing – review & editing, Formal analysis. **D. Davis:** Writing – review & editing, Project administration, Writing – review & editing, Formal analysis. **M.A. Shipolini:** Writing – review & editing, Data curation. **R. Magboo:** Writing – review & editing, Data curation. **F. Oezalp:** Writing – review & editing, Data curation. **V. Chester:** Writing – review & editing, Project administration.

## Ethical approval

The full protocol [10] and Clinical Investigation Plan is available elsewhere [https://clinicaltrials.gov/ct2/show/NCT06475703. Click or tap if you trust this link.">NCT06475703]. Ethical permissions were approved by the North of Scotland Research Ethics Committee 1 (24/NS/005) and the MHRA (CI/2024/0004/G).

## Data availability

To minimise the risk of disclosure of sensitive patient information, individual-level participant data are not publicly available. Access to a de-identified dataset (and/or aggregated outputs where individual-level sharing is not appropriate) may be provided to bona fide researchers on reasonable request. Requests should be directed to the Sponsor (R&D@gstt.nhs.uk) and will be assessed on a case-by-case basis, including the scientific rationale, feasibility, and privacy risks. Approval is subject to Sponsor governance processes, applicable data protection requirements, and execution of an appropriate data sharing agreement specifying permitted uses, retention, and security controls. Data will be made available through this route where approved.

## Funding sources

WISDOM is funded by the 10.13039/501100000272National Institute for Health and Care Research (10.13039/501100000272NIHR) under its 10.13039/501100009270Invention for Innovation funding stream (project reference NIHR204508). The views expressed are those of the authors and not necessarily those of the NIHR.

## Conflict of interest statement

J.J. is the co-founder of Isla Care Ltd. M.R. and K.C. are employed at GSTT. GSTT has a collaborative agreement with Isla Care Ltd.

## Figures and Tables

**figs1:**